# Development of a CRISPR-Cas9 System for Efficient Genome Editing of *Candida lusitaniae*

**DOI:** 10.1128/mSphere.00217-17

**Published:** 2017-06-21

**Authors:** Emily L. Norton, Racquel K. Sherwood, Richard J. Bennett

**Affiliations:** Department of Molecular Microbiology and Immunology, Brown University, Providence, Rhode Island, USA; Carnegie Mellon University

**Keywords:** CRISPR, DNA double-strand break, homology-directed repair, nonhomologous end joining

## Abstract

*Candida lusitaniae* is a member of the *Candida* clade that includes a diverse group of fungal species relevant to both human health and biotechnology. This species exhibits a full sexual cycle to undergo interconversion between haploid and diploid forms. *C. lusitaniae* is also an emerging opportunistic pathogen that can cause serious bloodstream infections in the clinic and yet has often proven to be refractory to facile genetic manipulations. In this work, we develop a clustered regularly interspaced short palindromic repeat (CRISPR) and CRISPR-associated gene 9 (Cas9) system to enable genome editing of *C. lusitaniae*. We demonstrate that expression of CRISPR-Cas9 components under species-specific promoters is necessary for efficient gene targeting and can be successfully applied to multiple genes in both haploid and diploid isolates. Gene deletion efficiencies with CRISPR-Cas9 were further enhanced in *C. lusitaniae* strains lacking the established nonhomologous end joining (NHEJ) factors Ku70 and DNA ligase 4. These results indicate that NHEJ plays an important role in directing the repair of DNA double-strand breaks (DSBs) in *C. lusitaniae* and that removal of this pathway increases integration of gene deletion templates by homologous recombination. The described approaches significantly enhance the ability to perform genetic studies in, and promote understanding of, this emerging human pathogen and model sexual species.

**IMPORTANCE** The ability to perform efficient genome editing is a key development for detailed mechanistic studies of a species. *Candida lusitaniae* is an important member of the *Candida* clade and is relevant both as an emerging human pathogen and as a model for understanding mechanisms of sexual reproduction. We highlight the development of a CRISPR-Cas9 system for efficient genome manipulation in *C. lusitaniae* and demonstrate the importance of species-specific promoters for expression of CRISPR components. We also demonstrate that the NHEJ pathway contributes to non-template-mediated repair of DNA DSBs and that removal of this pathway enhances efficiencies of gene targeting by CRISPR-Cas9. These results therefore establish important genetic tools for further exploration of *C. lusitaniae* biology.

## Abstract (precis)

The ability to perform efficient genome editing is a key development for detailed mechanistic studies of a species. *Candida lusitaniae* is an important member of the *Candida* clade and is relevant both as an emerging human pathogen and as a model for understanding mechanisms of sexual reproduction. We highlight the development of a CRISPR-Cas9 system for efficient genome manipulation in *C. lusitaniae* and demonstrate the importance of species-specific promoters for expression of CRISPR components. We also demonstrate that the NHEJ pathway contributes to non-template-mediated repair of DNA DSBs and that removal of this pathway enhances efficiencies of gene targeting by CRISPR-Cas9. These results therefore establish important genetic tools for further exploration of *C. lusitaniae* biology.

## INTRODUCTION

The *Candida* CTG clade is a collection of related species that translate CUG codons as serine instead of leucine, as in the universal genetic code (1). Several members of this clade are prevalent human pathogens, including *Candida albicans*, which is the most frequent cause of *Candida* bloodstream infections (2). The clade can be divided into two subclades consisting of haploid and diploid species, with diploid species generally being more common human pathogens than haploid species (1). Haploid species include those with important applications for biotechnology, such as the extremophilic yeast *Debaryomyces hansenii* (3).

*Candida lusitaniae* is a haploid member of the *Candida* clade and a human commensal fungus that infrequently causes fungemia, typically in patients with comorbidities (4–8). Mortality due to *C. lusitaniae* fungemia is 5 to 54% and is often attributed to its relatively high rates of resistance to amphotericin B, which are reported in 22 to 60% of clinical isolates (6, 9). Its emergence as a human pathogen may therefore be on the rise, similar to several other non-*albicans Candida* species (10), and multidrug resistance to both candins and azoles has recently been reported in clinical isolates (11). *C. lusitaniae* was also recently found at high levels in the lungs of cystic fibrosis patients, suggesting that this species has the potential to thrive in this niche (12). Furthermore, *Candida auris* is a newly emerging pathogen that is closely related to *C. lusitaniae* and is associated with multidrug-resistant infections in multiple countries (13, 14), making it even more relevant to understand how these *Candida* species develop antifungal resistance.

Research on *C. lusitaniae* has focused on its ability to undergo a complete sexual cycle, including mating, meiosis, and sporulation. Historically, all *Candida* species were labeled as obligate asexual species, and yet a number of these species have now been shown to undergo sexual or parasexual cycles (for reviews, see references 15 to 17). Reedy et al. established that *C. lusitaniae* is capable of a complete sexual cycle and that meiotic recombination is dependent on Spo11 (18), a topoisomerase-like factor that initiates meiotic recombination in diverse eukaryotes (19, 20). It is also clear that sexual reproduction in *C. lusitaniae* has been considerably rewired relative to other ascomycetes and that the programs regulating mating and meiosis have fused into one continuous sexual program (21). The retention of a full sexual program in *C. lusitaniae* is particularly striking given that this species has lost conserved components of the synaptonemal complex and the major crossover-formation pathway that acts in many other sexual species (1).

In order to better understand the biology of *C. lusitaniae*, it is necessary to develop a working genetic model. Historically, there have been several hurdles to the development or adoption of a functional model for *C. lusitaniae*, including its membership in the *Candida* CTG clade (10) as well as, in our experience, its relative intractability to genetic manipulation. However, the recent advent of the clustered regularly interspaced short palindromic repeat (CRISPR) and CRISPR-associated gene 9 (Cas9) system has revolutionized gene editing because of high-efficiency gene-targeting capabilities across diverse species (22). Two main CRISPR components are often combined for efficient mutagenesis: (i) a Cas9 endonuclease that catalyzes DNA double-strand break (DSB) formation and (ii) a single guide RNA (sgRNA) with a 20-bp protospacer specific to the target gene which guides Cas9 to the target locus. When Cas9 creates DSBs in the target gene, DNA can be repaired via nonhomologous end joining (NHEJ), resulting in mutations that disrupt gene function. Alternatively, homology-directed repair (HDR) can occur if extrachromosomal DNA is present that contains homology to regions close to the site of the DSB. In CRISPR systems, a deletion construct is often included in the transformation reaction to enable homologous recombination, as this can promote incorporation of a selectable marker and genotype analyses can easily determine if the marker has replaced the target gene.

Adaptations to CRISPR-Cas9 technology have allowed application of this system to the *Candida* clade. Vyas et al. developed a *Cas9* allele that is codon optimized for expression in *Candida* clade species and showed that CRISPR can successfully be used for genome editing in *Candida albicans* (23). Min et al. subsequently streamlined this approach so that CRISPR components can be expressed transiently rather than stably integrated into the *C. albicans* genome (24), and increased efficiencies were recently achieved by improving the expression of sgRNAs (25). In this study, we optimized the transient CRISPR system for efficient genetic manipulation of *C. lusitaniae*, a species recalcitrant to genome editing. We found that deletion frequencies were maximized using a deletion construct with long flanks (~1 kb) of homology together with CRISPR components that were expressed under the control of *C. lusitaniae*-specific promoters. In addition, we show that editing efficiencies could be further enhanced by deletion of NHEJ factors so that the majority of transformants often produced the desired gene deletions.

## RESULTS AND DISCUSSION

### An optimized CRISPR system for *C. lusitaniae.*

**FIG 1:**
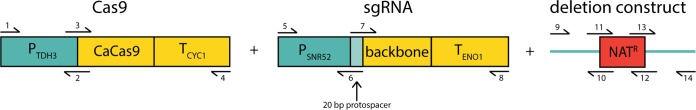
CRISPR components and targeting construct were optimized for transient CRISPR-Cas9 transformations in *C. lusitaniae*. Primers used to generate these constructs are shown, and their sequences are listed in Data Set S1 in the supplemental material. Cas9 was previously codon optimized for the *Candida* clade, CaCas9 (23), and the constitutive *C. lusitaniae TDH3* promoter ensured maximal expression. The single guide RNA (sgRNA), which enables Cas9 to identify the target gene, was composed of the *C. lusitaniae* constitutive *SNR52* promoter, a 20-bp protospacer sequence specific to the target gene, and the guide RNA backbone structure; terminator regions were included on both CRISPR components to help ensure efficient expression. Deletion constructs were also included in the transformation reaction to promote homology-directed repair of double-strand breaks created by Cas9. Two types of deletion constructs were generated by flanking a nourseothricin resistance marker (*caSAT1*) by either long (~1-kb) or short (~80-bp) regions of homology to the target gene. PCR construction of the long-flank deletion construct is shown in the figure; the short-flank deletion construct was generated using primers with ~80-bp homology to the target locus.

A transient CRISPR-Cas9 system was developed for *C. lusitaniae*, and its efficiency was determined for targeted deletion of the *ADE2* gene in haploid and diploid strains. This system does not rely on stable integration of CRISPR components into the genome but on their transient expression following transformation (24). A *Cas9* allele that has been codon optimized for the *Candida* clade (23) was placed under the control of the constitutive *C. lusitaniae TDH3* promoter (*ClTDH3*-driven Cas9 [ClCas9]; plasmid pRB732), and the single guide RNA (sgRNA) was expressed using the *C. lusitaniae* RNA polymerase III promoter from *SNR52* (Fig. 1) (plasmids pRB733 and -734; see Materials and Methods). We compared the efficiency of this *C. lusitaniae*-optimized system to that in which *Cas9* was expressed using the constitutive *C. albicans ENO1* promoter (CaCas9), and sgRNA expression was driven by the* C. albicans SNR52* promoter (23) (plasmids pRB736 and -737). For both expression systems, we used the same CTG codon-optimized *Cas9* sequence. Two guide RNAs with different 20-bp protospacers were used to target sites within the *C. lusitaniae ADE2* gene (*CLUG_04693*). We targeted *ADE2* since mutants lacking this gene develop a distinctive red-colony phenotype instead of the normal white phenotype, providing a visual readout of loss of function. To test the effect of length of the homologous flanks on the efficiency of gene targeting, we compared two gene deletion constructs: a “short-flank” deletion construct with 80 bp of homology flanking a nourseothricin resistance marker (*caSAT1*) and a “long-flank” deletion construct that had ~1-kb homologous flanks (plasmid pRB620). Finally, to determine how each component of the CRISPR system affects targeting efficiency, individual components were systematically omitted from transformation reaction mixtures. We note that distinct guide RNAs were used for haploid and diploid strains, so that the results between these transformations are not directly comparable. However, the same combination of CRISPR-Cas9 components produced the highest transformation efficiencies in both haploid and diploid strains tested in this study.

10.1128/mSphere.00217-17.2 DATA SET S1  Strains, plasmids, and oligonucleotides used in this study. Download DATA SET S1, XLSX file, 0.02 MB. Copyright © 2017 Norton et al. 2017 Norton et al. This content is distributed under the terms of the Creative Commons Attribution 4.0 International license.

### Analysis of CRISPR transformations.

**FIG 2:**
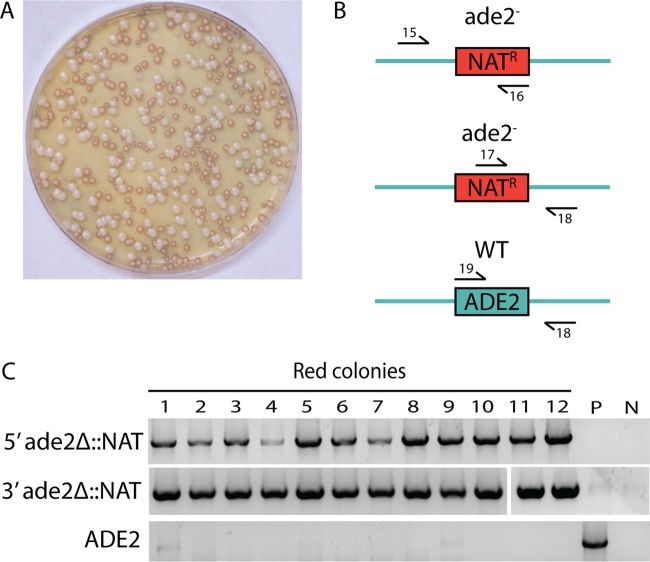
Genotypic analysis of red colonies arising from CRISPR-Cas9 targeting of *ADE2* in a *C. lusitaniae* haploid strain. (A) A *C. lusitaniae*-optimized CRISPR-Cas9 system was used to target the *ADE2* locus, and a large percentage of red-colony (*ade*^−^) phenotypes was observed (gamma of image adjusted to emphasize red/white color differences). (B) PCR assays for identifying *ade2* genotypes, with arrows indicating the relative positions of the primers used (see Data Set S1 for sequences). WT, wild type. (C) For all 12 red colonies shown here, the 5′ and 3′ junction checks were positive and the ORF checks were negative, indicating successful replacement of the *ADE2* target gene with the nourseothricin resistance marker. The parent strain (RYS284) was used as a wild-type control (P), and template DNA was omitted in the negative control (N).

*C. lusitaniae* transformations were performed essentially as described previously (21), in which genetic constructs were introduced into cells by electroporation (see Materials and Methods). All transformation selections utilized the *caSAT1* gene (26), which supports growth of *C. lusitaniae* cells on nourseothricin (18). Colonies were counted 48 to 72 h after plating to the selection medium (nourseothricin-resistant [NAT^r^] colonies) and then further monitored for 7 to 10 days to allow red pigmentation to develop in *ade2* mutant colonies (Fig. 2A). Red colonies were further verified as being *ade*^−^ by patching to plates lacking adenine (Ade^−^ medium), on which none of the red colonies were able to grow (*n =* 195). Additionally, we conducted PCR genotype analyses to determine the presence or absence of the *SAT1* marker or the *ADE2* open reading frame (ORF) at the native *ADE2* locus (Fig. 2B). In haploid strains, the *SAT1* targeting construct accurately replaced the *ADE2* gene in all of the red colonies examined (36/36) (Fig. 2C). In diploid strains, the majority of red colonies (34/36) also contained the *SAT1* marker successfully integrated at the native* ADE2* locus as well as the absence of the *ADE2* ORF. However, 2 out of 36 red colonies in the diploid showed evidence for accurate integration of *SAT1* at the *ADE2* locus and yet still showed the presence of the *ADE2* ORF, and these colonies are further discussed below. Together, these results indicate that CRISPR-mediated DSBs can be efficiently repaired by a homology-directed repair strategy in both haploid and diploid strains.

Gene-targeting efficiencies were compared using the various combinations of DNA constructs shown in Table 1, and experiments were performed in triplicate for both haploid and diploid strains. Note that in all cases only the gene-targeting construct was selected for using nourseothricin and that the other components are therefore presumed to be transiently expressed in the cell, as previously shown for *C. albicans* (24). We did not check for ectopic integration of any CRISPR components in these experiments, although this could be examined in future studies. In comparing different approaches, there were several notable trends observed:

**TABLE 1:** Transformation efficiencies of a transient CRISPR-Cas9 system targeting *ADE2* in *C. lusitaniae* haploid and diploid strains

CRISPR component			Mean (SD) for strain type:			
			Diploid^c^		Haploid^e^	
Deletion construct^a^	Cas9^b^	sgRNA^b^	No. of NAT^r^ colonies	% *ade2*^d^	No. of NAT^r^ colonies	% *ade2*^d^
Long	Clus	Clus	520 (107)	7.9 (3.5)	801 (495)	36 (2)
	Calb	Calb	211 (248)	0	281 (244)	17.0 (13)
	—	Clus	282 (359)	0.6 (0.8)	341 (220)	21.0 (17)
	Clus	—	418 (285)	0.0 (0.1)	879 (225)	3.1 (3.0)
	Calb	—	82 (112)	0.2 (0.3)	335 (108)	14.0 (9)
	—	—	228 (281)	0.4 (0.7)	231 (71)	8.4 (9.7)
Short	Clus	Clus	14 (19)	0	41 (40)	2.3 (3.1)
	Calb	Calb	8.0 (13.0)	0	76 (68)	0
	—	Clus	13 (14)	0	26 (14)	0
	Clus	—	9.3 (14.5)	0	18 (14)	0
	Calb	—	1.0 (1.7)	0	37 (22)	0
	—	—	1.7 (2.9)	0	22 (6)	0

a Deletion constructs had long flanks (~1-kb homology both 5′ and 3′ to the *C. lusitaniae ADE2* gene) or short flanks (~80-bp homologous flanks).

b “Clus” and “Calb” indicate that the Cas9 and single guide RNA (sgRNA) are under a *C. lusitaniae* or *C. albicans* promoter, respectively. The 20-bp protospacer in the sgRNA was different between the haploid and diploid strains. Dashes indicate that a component was not included in the transformation.

c Diploid strain, *C. lusitaniae* CAY5019 (*n =* 3 transformations).

d *ADE2* deletion percentage, number of red colonies/total number of NAT^r^ colonies × 100.

e Haploid strain, *C. lusitaniae* RSY284 (*n* = 3 transformations).

First, the use of deletion constructs with longer-homology flanks (~1 kb) significantly increased both the total number of NAT^r^ transformants and the fraction of successful transformants compared to constructs with shorter-homology flanks (~80 bp). This was true for transformations that included the CRISPR-Cas9 system and for those that did not. For example, in the absence of CRISPR, diploid cells transformed with the short-homology *ADE2*-targeting construct gave an average of 1.7 NAT^r^ colonies per transformation, none of which were adenine deficient (Table 1). Transformations with the longer-homology construct gave an average of 228 colonies per transformation of diploid cells, an increase of >100-fold, although targeting efficiency remained very low, with only 0.4% of these colonies being adenine deficient. Similar trends were observed with haploid *C. lusitaniae* cells, with the longer-homology construct generating >10-fold as many NAT^r^ transformants as the shorter-homology construct. The increase in homology also successfully produced *ade*^−^ cells in 8.4% of haploid transformants even in the absence of CRISPR, whereas none of the haploid transformants obtained with shorter-homology flanks were *ade*^−^ (Table 1).

Second, we observed that CRISPR-Cas9 significantly enhanced the efficiency of gene targeting in *C. lusitaniae*. We compared two versions of CRISPR-Cas9: one contained constructs previously used for gene targeting in *C. albicans* (23) and a second contained modified Cas9 and sgRNA constructs engineered to be under the control of *C. lusitaniae* promoters. Notably, the latter resulted in a large increase in targeting efficiencies compared to the use of the more generic *C. albicans* CRISPR-Cas9 system or non-CRISPR controls. Thus, in the diploid strain, the use of *C. lusitaniae*-optimized CRISPR-Cas9 with an *ADE2*-targeting construct (and long 1-kb flanks) generated both the highest number of NAT^r^ transformants and the highest frequency of *ade2* mutants (on average, we obtained 520 NAT^r^ colonies, of which 7.9% were *ade2* mutants [Table 1]). This reveals that the *C. lusitaniae-*optimized CRISPR-Cas9 system increased the number of transformants by more than 2-fold and the targeting efficiency by 20-fold relative to controls without CRISPR components. In contrast, we found that the *C. albicans* CRISPR-Cas9 system did not enhance transformation efficiencies relative to controls, as both the number of transformants and the frequency of *ade*^−^ colonies were comparable to those in experiments lacking CRISPR (Table 1). This illustrates that it can be critically important to utilize constructs whose expression is tailored to the *Candida* species being studied.

Similar trends were observed in the haploid strain, where the efficiency of obtaining *ADE2* gene deletions was higher than that in diploid cells. An average of 36% of haploid NAT^r^ colonies were *ade*^−^ using the *C. lusitaniae-*optimized CRISPR system together with the longer-homology deletion construct (Table 1). Several successful transformants were obtained in haploid cells using this deletion construct even in the absence of CRISPR (8.4% *ade*^−^ transformants). A lower targeting efficiency was observed using the short-homology deletion construct with CRISPR (2.3% of transformants were *ade*^−^), whereas this deletion construct produced no *ade*^−^ transformants in the absence of CRISPR (Table 1).

We were further able to dissect the contribution of each of the components of the CRISPR system by performing transformations that were lacking either Cas9 or sgRNA. Thus, whereas transformation of diploid cells with the full *C. lusitaniae*-optimized CRISPR system (and a deletion construct with long-homology flanks) successfully deleted the *ADE2* genes in 7.9% of selected transformants, removal of either Cas9 or sgRNA significantly lowered targeting efficiencies to 0.6% and 0%, respectively (Table 1). These numbers are comparable to those for transformations lacking both Cas9 and sgRNA (0.4% efficiency) and establish that the entire CRISPR-Cas9 system is necessary for efficient gene targeting. Similar results were obtained in haploid cells, where both Cas9 and sgRNA were necessary for optimal gene-targeting efficiencies (Table 1).

### Homologous versus nonhomologous DNA repair.

Repair of DSBs is thought to occur via competing pathways involving either homology-directed repair (HDR) or nonhomologous end joining (NHEJ) (27). While we selected for transformants that had used HDR to incorporate gene-targeting templates in the experiments described above, it is also possible for CRISPR-mediated DSBs to be processed by the NHEJ pathway, resulting in ORF-disrupting mutations at the junction site (28). We therefore conducted the following experiments to investigate the contribution of NHEJ to the repair of CRISPR-derived DSBs in *C. lusitaniae*.

We first compared the efficiency with which the *C. lusitaniae*-optimized CRISPR system disrupted the *ADE2* gene in wild-type strains versus those lacking NHEJ. To do this, *C. lusitaniae* strains were constructed lacking *KU70* (*CLUG_03491*) and/or *LIG4* (*CLUG_01056*), two genes that have been intimately associated with the canonical NHEJ pathway in eukaryotic species (27). The Ku70/80 complex is required for the recognition of DSBs, whereas a complex containing ligase 4 performs the joining of DNA ends during NHEJ (for reviews, see references 27, 29, and 30). Disruption of these genes can reduce NHEJ and thereby promote recombination by HDR in multiple fungal species, including the related *Candida* clade species *Candida guilliermondii* (31, 32), although the role of NHEJ has not been investigated in *C. lusitaniae*.

We found that haploid *C. lusitaniae* strains lacking NHEJ genes enhanced integration of the *SAT1* marker at the *ADE2* locus by HDR, so that the frequency of targeting was 2 to 3 times higher than that in the control strain. Thus, whereas 25% of CRISPR-generated NAT^r^ colonies were *ade*^−^ in the control strain, this increased to 49% of NAT^r^ colonies in a *ku70* mutant and to 81% of NAT^r^ colonies in a *ku70 lig4* double mutant (Table 2; *P* = 0.03 for the *ku70 lig4* mutant compared to control, one-tailed *t* test). This improvement is similar to that seen in *Aspergillus nidulans*, where deletion of the *KU70* homolog increased the frequency of correct gene targeting from 38% to 89% (using 500-bp-homology flanks [33]). Our results suggest that the NHEJ repair pathway may be responsible for processing a subset of the DSB events that CRISPR-Cas9 induces at the target locus. In addition, removal of NHEJ presumably reduces nonspecific integration of the *SAT1* marker at ectopic sites in the genome. Thus, the use of NHEJ-defective strains can improve gene targeting in *C. lusitaniae*, and this could be particularly beneficial if genes are recalcitrant to deletion by the standard CRISPR-Cas9 approach.

**TABLE 2:** Analysis of CRISPR-Cas9 efficiencies in wild-type and NHEJ mutant backgrounds^a^

Strain	Mean (SD)	
	No. of NAT^r^ colonies	% *ade2*
RSY426 (*ku70 lig4*)	531 (514)	81 (17)
RSY376 (*ku70*)	306 (200)	49 (37)
RSY281 (parent haploid)	1,240 (1,144)	25 (32)

a For this experiment, deletion constructs had long flanks (~1-kb homology flanking the *ADE2* gene). *n =* 3 transformations per strain. The Cas9 and sgRNA are under a *C. lusitaniae* promoter.

We also performed transformations using the *C. lusitaniae*-optimized CRISPR system in the absence of the *ADE2* targeting construct. Any *ade*^−^ transformants produced by this experiment should therefore arise via mutagenesis during NHEJ processing. Following transformations, cells were plated to nonselective medium (yeast extract-peptone-dextrose [YPD]), and colonies were analyzed for white or red phenotypes. We observed only white colonies in three independent experiments for haploid and diploid strains (2,391 colonies analyzed for haploid strain RSY284 and 3,195 colonies analyzed for diploid strain CAY5019). This indicates that the level of mutagenesis at the *ADE2* locus induced by the CRISPR-Cas9 system is not sufficiently high to detect these events without selection.

**FIG 3:**
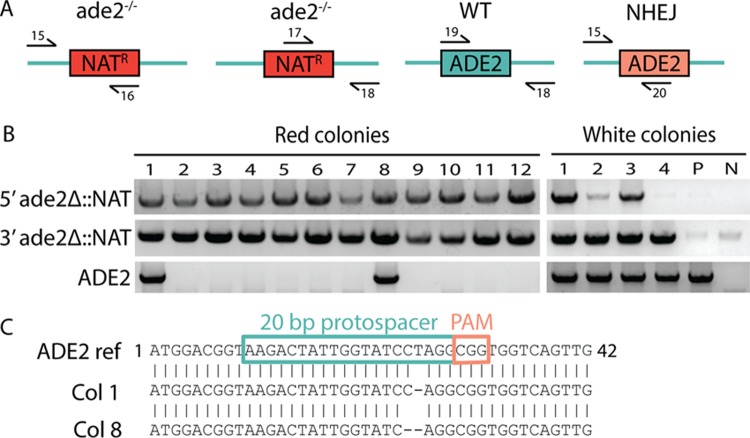
Genotypic analyses and sequencing of *C. lusitaniae* diploid transformants. (A) PCR assays for identifying *ade2* or wild-type (WT) genotypes, with arrows indicating the relative positions of the primers used (see Data Set S1 for sequences). (B) Results of genotypic analyses for red and white diploid colonies, indicating that most red colonies exhibit replacement of *ADE2* with the *SAT1* gene. However, two red colonies (colonies 1 and 8) had positive *SAT1* junction checks but still contained the *ADE2* ORF, indicating that one allele was repaired via homologous recombination and the other was repaired via NHEJ. P, parent strain (CAY5019); N, negative control (no DNA). (C) Sequencing of the *ADE2* ORF in colonies 1 and 8 shows that mutagenesis via NHEJ resulted in a 1- or 2-nucleotide deletion within the protospacer preceding the PAM sequence (red box).

Finally, we further examined the genotypes of two *ade*^−^ colonies obtained following CRISPR-mediated transformation of diploid cells that gave positive PCR results both for the presence of *SAT1* at the *ADE2* locus and for the continued presence of the *ADE2* ORF (strains CAY8421 and -8422). We considered that one *ADE2* allele had been replaced via HDR while the other may have been mutagenized via NHEJ. To test this, the *ADE2* ORF in these strains was PCR amplified and sequenced. We found that in both cases the remaining *ADE2* allele contained a frameshift mutation within the region targeted by the protospacer present in the sgRNA (Fig. 3). The two *ade*^−^ diploids contained deletions of 1 or 2 bp at the same position, resulting in distinct frameshift mutations. These results establish that NHEJ was used to repair one of the two *ADE2* alleles following a CRISPR-generated DSB and introduced errors that led to loss of *ADE2* function.

### CRISPR-mediated targeting of other *C. lusitaniae* genes.

To explore the ability of CRISPR-mediated transformations to target other loci in *C. lusitaniae*, we performed experiments to delete three additional genes in haploid and diploid strains. These were *UME6* (*CLUG_03546*) and *REC8* (*CLUG_00439*), encoding orthologs of factors involved in meiosis in *Saccharomyces cerevisiae*, and *MTLalpha1* (*CLUG_04923*), encoding a transcription factor located at the *MTLalpha* locus. When *UME6* was targeted for deletion using *C. lusitaniae*-optimized CRISPR-Cas9, an average of 19% and 81% of PCR-checked NAT^r^ colonies from the diploid (RSY432) and the haploid (RSY284/RSY411) transformations showed successful replacement of the *UME6* ORF with the *SAT1* gene, respectively (diploid, *n =* 3 transformations, 126 colonies tested; haploid, *n =* 2 transformations, 20 colonies tested). Interestingly, although there is only one copy of *MTLalpha1* in both the haploid α strain and diploid **a**/α strain, the efficiency of deleting this gene was less than half that for *ADE2* or *UME6* (diploid, *n =* 2 transformations, 48 colonies analyzed, 9.4% of NAT^r^ colonies were *alpha1* deletion mutants; haploid, *n =* 3 transformations, 88 colonies analyzed, 18% of NAT^r^ colonies were *alpha1* mutants).

We found that we were initially unable to successfully delete *REC8* in wild-type haploid (RSY411) or diploid (RSY432) *C. lusitaniae* strains (*n =* 2 transformations each, 58 colonies tested). However, when we performed transformations in the haploid *ku70 lig4* double mutant strain (RSY426), 69% of NAT^r^ colonies were *rec8* mutants (*n =* 1 transformation, 13 colonies tested). This result indicates that strains defective in NHEJ can allow for CRISPR targeting of genes that are recalcitrant to deletion in wild-type strain backgrounds.

Finally, we attempted to delete two genes simultaneously by using CRISPR-Cas9 and including guide RNAs and long-homology deletion constructs to target both *ADE2* and *UME6* loci in one transformation (haploid strains RSY281, RSY284, and RSY426). We found that 0 to 28% of NAT^r^ colonies were double mutants that had accurately replaced both *ADE2* and *UME6* genes (*n =* 4 transformations, 24 red [*ade*^−^] colonies tested for each). While these results reflect considerable variability in the targeting of different loci with the CRISPR system, we emphasize that it is possible to use this system to generate deletions for multiple genes simultaneously in *C. lusitaniae*.

### Concluding remarks.

The use of CRISPR-Cas9 has transformed our ability to edit genome sequences from bacteria to humans. Here, we describe a species-specific version of CRISPR-Cas9 that has been modified from that developed for the related *Candida* clade species *Candida albicans* (23, 24). Our results highlight the necessity of modifying this system and the use of species-specific promoters to drive expression of CRISPR-Cas9 components for achieving high transformation efficiencies in *C. lusitaniae*. We show that CRISPR can be used to target multiple genes and that transformation efficiencies for template-directed repair are further increased upon deletion of the NHEJ pathway in *C. lusitaniae*. As an alternative approach, Hogan and colleagues have utilized the transformation of RNA-protein complexes that allow species-independent CRISPR-Cas9 editing of *Candida* genomes (see accompanying paper by Grahl et al. [34]). There are therefore now two distinct CRISPR-Cas9 methodologies that support efficient genome editing in *C. lusitaniae*.

## MATERIALS AND METHODS

### Strains and media.

*C. lusitaniae* strains RSY284 (haploid **a**, *ura3*), RSY286 (haploid α, *leu2*), RSY147 (haploid **a**, *chx*^r^), RSY432 (diploid **a**/α, *ARG4/arg4*::*FRT ADE2/ade2*::*FRT URA3/ura3 chx*^S^/*chx*^r^) and RSY411 (haploid α, *arg4*::*FRT ade2*::*FRT chx*^r^) were used in this study (see Data Set S1 in the supplemental material). Strains RSY284 and RSY286 were mated to produce CAY5019 (diploid **a**/α, *URA3/ura3 LEU2/leu2*). All strains were grown in YPD (2% Bacto peptone, 2% glucose, 1% yeast extract, 25 µg/ml uridine) at 30°C with shaking, and transformants were selected on YPD supplemented with 200 µg/ml nourseothricin (YPD plus NAT).

### NHEJ plasmids and strain construction.

*C. lusitaniae* strain RSY147 was used to generate a *SAT1*-recycled *leu2*^−^ strain (RSY281, haploid **a**, *leu2*), using homologous recombination of a *leu2* deletion plasmid (pRB310) linearized with ApaI and SacII restriction enzymes (see Data Set S1 for primer sequences). *KU70* and *LIG4* deletion constructs were generated by PCR amplifying and cloning ~950-bp *C. lusitaniae* 5′ and 3′ homologous flanks into the pSFS2a plasmid (26) containing the nourseothricin resistance marker (plasmids pRB254 [*KU70*] and pRB247 [*LIG4*]; see Data Set S1 for primer sequences). Plasmids were linearized with KpnI and SacII restriction enzymes prior to transformation. *C. lusitaniae* strain RSY281 was used in electroporation transformations to delete *KU70* (strain RSY376) and subsequently delete *LIG4* (strain RSY426).

### CRISPR plasmids and DNA constructs.

The CTG clade codon-optimized Cas9 (CaCas9) and guide RNA were PCR amplified from plasmid pV1093 (23) (see primers in Data Set S1). To optimize Cas9 and guide RNA expression in *C. lusitaniae*, the *C. albicans* promoters were replaced with *C. lusitaniae* constitutive *TDH3* and *SNR52* promoters, respectively (plasmids pRB732 to -734). The *TDH3* locus for *C. lusitaniae*, *CLUG_03499*, is annotated in the Candida Genome Database (CGD) (35). The *C. lusitaniae SNR52* locus was identified using the BLASTN tool on CGD to obtain the region of the *C. lusitaniae* genome with the greatest homology to the *C. albicans SNR52* gene. For *TDH3* and *SNR52*, the upstream ~1-kb regions were PCR amplified from *C. lusitaniae* genomic DNA, and fusion PCR was used to attach these fragments to the Cas9 and sgRNA scaffolds (see primers in Data Set S1). sgRNAs were designed to target *C. lusitaniae ADE2*, *UME6*, *MTLalpha1*, and *REC8* genes by using 20-bp protospacers immediately followed by a PAM sequence of the structure NGG (or preceded by CCN if located on the reverse strand). Protospacers were identified based on previously established criteria (23), but we considered only candidates located within the first half of the genes so that mutations would more likely generate nonfunctional proteins. We also selected sgRNAs that had minimal off-target effects identified by cross-referencing the 12 nucleotides (nt) proximal to the PAM sequence against the *C. lusitaniae* genome using NCBI download *Clavispora lusitaniae* ATCC 42720 (assembly ASM383v1). By using oligonucleotide primers containing the unique 20-bp protospacer sequence plus a 20- to 30-bp overlap with the upstream or downstream sequence, we amplified the sgRNA in two fragments; these fragments were then stitched together by fusion PCR to integrate the new protospacer (see primers in Data Set S1). Deletion constructs were generated with either long (~1-kb) or short (80-bp) homologous flanks. Long-flank deletion constructs were generated using fusion PCR of 1-kb upstream and downstream flanking regions plus ~1.8 kb of the *SAT1* marker, which were then cloned into a pCR-Blunt II-TOPO vector (*ADE2*, pRB620; *UME6*, pRB744; *MTLalpha1*, pRB768; *REC8*, pRB748). Short-flank deletion constructs were generated by PCR amplifying the ~4.3-kb *SAT1* marker and flipper cassette with long oligonucleotides that contained ~80 bp of the upstream and downstream flanking regions of the target gene. PCRs (50-µl mixtures) were conducted with Phusion enzyme (Thermo Scientific) to generate all DNA constructs (1× Phusion HF buffer, 200 µM deoxynucleoside triphosphates [dNTPs], 0.2 µM [each] primer, ~20 to 50 ng template DNA, and 1 unit Phusion DNA polymerase; run according to the manufacturer’s recommendations). To stitch constructs together by fusion PCR, the products from prior Phusion PCRs were combined and diluted 10-fold, and then 1 µl was used as a template in the fusion PCR.

### *C. lusitaniae* transformations.

An electroporation transformation protocol used for both haploid and diploid strains was adapted from reference 21. (i) Overnight cultures were diluted to an optical density at 600 nm (OD_600_) of 0.4 in liquid YPD and then grown until they reached a final OD_600_ of 1.5 to 1.7. (ii) Cells were pelleted and resuspended in 10 ml of transformation buffer composed of 0.1 M lithium acetate (LiOAc), Tris-EDTA, pH 8.0, and 0.01 M dithiothreitol. (iii) Cells were incubated on a shaker at 22°C for 1 h, washed with ice-cold water, and resuspended in ice-cold 1 M sorbitol to acquire a concentration of 150 OD units/ml. (iv) Forty microliters of cells was combined with 3 µg deletion construct, 1 µg Cas9 construct, and/or 1 µg sgRNA construct (Table 1) in 0.2-cm electroporation cuvettes (Bio-Rad, Hercules, CA). (v) Cells were electroporated at 1.8 kV, 200 Ω, and 25 µF for 4.5 to 5 ms (Bio-Rad MicroPulser); immediately resuspended in 1 ml YPD; and allowed to recover overnight at 30°C. (vi) Cells were plated onto selective medium (YPD plus NAT) to identify successful transformants.

10.1128/mSphere.00217-17.1 TEXT S1 Sequences of constructs and guides used in this study. Download TEXT S1, DOCX file, 0.02 MB. Copyright © 2017 Norton et al. 2017 Norton et al. This content is distributed under the terms of the Creative Commons Attribution 4.0 International license.
