# Supplementary material for: Development of a CRISPR-Cas9 System for Efficient Genome Editing of Candida lusitaniae
Source: mSphere. 2017 Jun 21;2(3):e00217-17. doi: 10.1128/mSphere.00217-17 (PMC5480034; doi:10.1128/mSphere.00217-17)
Supplement: TEXT S1 [file sph003172301s2.docx]

**Supplemental Sequences**

ClCas9 (including Cl TDH3 promoter, CaCas9, and CYC1 terminator; 5562 bp; pRB732):

GGAAAGCAAGCAATCGTAGCCCCAGCAAAGCACTATAGTAGGTGTTTGCTCTTTTTATTTCCTTTGCTCTACGCACTGTAATACAGGATTGATGAAAGCAGTATAGAAAATCGCCTCGAAGCCTCGTGAATCAGTAGATCTATTTACATTGTTCGTCTCGTGCGGTTTTTCACCAGTGTCATGAGCGAGCGAACTTGGTCGACATCATTAAAAAAAACTGCGGCTTTCGTGTCAGAGTCGCTGTGTATCACTTCGACGTAGGTAATCAGCTCTTAAGCTTGAAGTGTGGATCGGAATTTTCTTCTCCTCCTTCATTAGTCATCTTCCATGAAAGAGTGACTTTTGACTCACACGAAGGAAAATGTGCGCAAATTACAAATCACCTACAAGCCACAAGACACATTCATCAAAGGCACCAAACAAGACATTCAAGTGGACCATGATAAAAGCCAAGGTTTGAAGGCATATTCTGTATACTTCAATCTTGGTTTGCTCGGCCCAAGTCCCGGCGATTAGCGGCGCCCGGTGGCCCGGCTGGATAGATTTCAATTGGCACACGCACAATTATCTCCACAATTGCATTGCATACACCTTTACACCACAACTCGGCGACCTCGGCCAGCCTCTGAAAACACATAGCCAAAAACGAAGAAGGGACAGGCATTTTCCCTTCATTGCCTAGTATGGCATAATACGTCACCAAACCATGCATTATGGATTGTTAAGAGCTAATCTTCCTGGTGAAAAACCGCCACAAATTGACCATGACAGTGCTTGCTCGGAGTTTTCAGCGGAGGGCGTGCGCCCGGGCAAACATAGTGCATTGTGATGTAGGCGGCAAATATATGCCGGATACGCGCTTATGGAGCGGATTGCCCCGGCGGAGAAAAGTATATAAGTAGCCAGCGCAAGGCCCCCATTTCTAGAGAATTTTTCCCTTCAATTGATTACAACTTCAATTAACTACAAACAAAACTATCAATCATGGATAAAAAGTATAGTATTGGTTTAGATATTGGTACTAACTCTGTGGGTTGGGCAGTTATCACCGACGAATATAAAGTTCCATCAAAGAAATTTAAGGTGTTAGGTAACACTGACAGACACTCAATAAAAAAGAATCTTATCGGTGCTCTTTTGTTCGACTCCGGTGAAACTGCCGAGGCTACACGTTTAAAAAGAACAGCAAGAAGAAGATATACCCGTAGAAAAAATAGAATATGTTATTTACAAGAAATCTTTTCTAATGAAATGGCTAAAGTTGATGATTCCTTTTTCCATAGATTGGAAGAGTCATTTTTGGTTGAAGAAGACAAAAAGCATGAGAGACATCCAATCTTTGGGAATATAGTTGATGAAGTGGCTTACCATGAAAAATATCCTACCATTTATCATTTAAGAAAGAAATTGGTAGATTCAACTGATAAAGCTGACCTTAGATTAATCTATTTAGCACTTGCCCATATGATTAAATTTAGAGGTCATTTTTTGATTGAAGGTGATTTGAACCCAGATAATTCTGACGTGGATAAATTATTTATTCAATTAGTCCAAACCTACAACCAATTATTTGAGGAAAATCCAATTAATGCTAGTGGTGTCGATGCCAAAGCTATATTATCAGCCAGATTATCAAAATCTAGACGTTTGGAAAATTTGATTGCCCAATTGCCAGGAGAAAAAAAGAATGGATTATTTGGAAACTTGATCGCATTATCATTGGGTTTGACACCAAATTTTAAATCTAATTTTGATTTAGCTGAAGATGCTAAATTACAATTATCAAAAGACACCTATGACGACGATTTGGACAATTTACTTGCTCAAATTGGTGATCAATATGCAGATTTGTTCTTAGCTGCTAAAAACTTATCTGATGCTATTTTGTTGTCTGATATTTTGAGAGTGAACACAGAAATAACCAAAGCTCCATTATCAGCATCTATGATCAAACGTTATGATGAACACCATCAGGATTTGACTTTATTGAAAGCTTTGGTGAGACAACAATTGCCAGAGAAGTATAAAGAAATCTTTTTCGATCAATCTAAAAACGGGTATGCAGGTTATATTGATGGGGGTGCCTCCCAAGAGGAATTTTACAAATTTATAAAACCTATTTTAGAAAAGATGGATGGGACTGAGGAACTTTTGGTCAAATTGAACAGAGAAGATTTGTTACGTAAACAGAGAACTTTTGATAATGGTAGTATACCTCACCAAATTCATTTGGGTGAGTTGCATGCAATTTTAAGAAGACAAGAAGATTTTTATCCATTTTTAAAAGATAATAGAGAAAAAATCGAGAAAATTTTAACCTTTAGAATTCCATACTATGTTGGGCCTTTGGCTAGAGGTAATTCAAGATTTGCCTGGATGACACGTAAATCAGAAGAAACTATTACCCCTTGGAATTTTGAAGAGGTTGTTGATAAAGGAGCATCAGCACAGAGTTTTATTGAAAGAATGACCAATTTCGATAAAAACTTACCAAATGAAAAAGTTTTACCAAAACATTCCTTGTTATACGAATATTTTACTGTTTACAATGAACTTACAAAGGTTAAATATGTTACTGAAGGTATGCGTAAGCCAGCCTTTTTATCTGGAGAACAGAAAAAGGCAATAGTTGATTTATTGTTTAAAACAAATAGAAAAGTTACTGTTAAACAATTAAAAGAAGATTACTTTAAGAAAATTGAATGTTTTGATTCAGTTGAAATCAGTGGTGTTGAAGACAGATTTAATGCTAGTTTAGGAACTTACCATGATTTACTTAAAATTATCAAAGATAAAGATTTCTTGGATAACGAAGAAAATGAAGACATTTTAGAAGACATTGTTTTAACCTTAACTTTATTCGAAGATAGAGAGATGATTGAAGAACGTTTGAAGACTTATGCACATTTGTTTGACGATAAAGTGATGAAACAGTTGAAAAGAAGACGTTATACTGGATGGGGTAGATTGTCTCGTAAATTGATCAATGGAATTAGAGATAAACAAAGTGGTAAAACTATCTTGGACTTTTTGAAATCTGACGGATTTGCTAATAGAAATTTCATGCAATTGATCCACGACGATAGTTTGACATTTAAAGAAGACATCCAAAAGGCCCAAGTGAGTGGGCAAGGTGATTCATTACATGAACATATTGCAAATTTAGCCGGATCTCCTGCTATTAAGAAAGGGATATTACAAACTGTTAAAGTTGTGGATGAATTAGTGAAAGTAATGGGAAGACATAAACCTGAAAACATTGTCATTGAGATGGCAAGAGAAAATCAAACTACACAAAAAGGACAGAAAAATAGTAGAGAACGTATGAAAAGAATAGAAGAGGGTATTAAAGAATTGGGTAGTCAAATATTGAAAGAACACCCAGTGGAAAATACCCAGTTGCAAAATGAAAAATTATATCTTTACTACCTTCAAAATGGACGTGATATGTATGTTGATCAGGAATTAGATATAAATAGACTTTCAGATTATGATGTAGATCATATAGTTCCACAATCTTTCTTGAAAGATGATTCCATAGACAATAAAGTATTAACTAGAAGTGATAAAAATAGAGGTAAAAGTGATAATGTCCCAAGTGAGGAAGTCGTCAAAAAGATGAAAAATTACTGGCGTCAACTTTTGAATGCTAAATTAATTACTCAAAGAAAATTTGATAATTTGACTAAAGCAGAAAGAGGTGGGCTTTCTGAATTAGATAAAGCCGGGTTCATTAAAAGACAATTGGTCGAAACTAGACAAATTACTAAACATGTTGCCCAAATTTTAGATTCCCGTATGAACACTAAGTATGACGAAAATGATAAGTTAATACGTGAGGTTAAAGTCATTACTTTAAAATCAAAACTTGTCTCTGATTTCAGAAAGGATTTCCAATTCTATAAAGTTAGAGAAATTAATAATTATCATCATGCTCATGATGCATATTTGAATGCTGTAGTTGGAACTGCTTTAATCAAGAAATACCCTAAATTAGAATCTGAATTTGTATATGGTGATTACAAAGTCTATGATGTTAGAAAGATGATTGCTAAATCAGAACAAGAAATTGGTAAAGCTACAGCTAAATACTTCTTTTACTCTAACATTATGAATTTCTTTAAAACAGAAATTACTTTGGCAAACGGTGAAATTAGAAAAAGACCTCTTATTGAAACAAATGGTGAGACTGGAGAGATAGTTTGGGACAAAGGGCGTGATTTCGCTACTGTTAGAAAAGTTTTATCAATGCCACAAGTTAACATTGTAAAGAAAACAGAGGTTCAAACTGGTGGTTTCTCAAAAGAAAGTATTTTGCCTAAAAGAAATAGTGATAAATTGATTGCCAGAAAAAAGGATTGGGATCCAAAGAAATATGGTGGTTTCGACTCACCAACCGTAGCCTATTCTGTTTTGGTTGTGGCAAAGGTTGAAAAGGGTAAAAGTAAAAAGCTTAAATCAGTAAAAGAACTTTTGGGTATTACAATAATGGAAAGAAGTTCCTTTGAAAAGAACCCTATTGATTTTTTGGAAGCTAAAGGTTATAAGGAAGTAAAGAAGGACTTAATAATCAAATTGCCTAAATATTCTTTATTTGAATTAGAAAATGGGAGAAAAAGAATGTTGGCTTCTGCTGGAGAATTGCAAAAGGGTAATGAATTAGCATTGCCTTCCAAATATGTTAACTTCTTGTATTTAGCTTCACACTATGAAAAGTTGAAAGGGTCACCAGAAGATAACGAGCAAAAACAATTATTTGTTGAACAACACAAACACTACTTAGATGAGATTATAGAACAAATTAGTGAATTCAGTAAAAGAGTGATATTAGCTGATGCAAATTTAGATAAAGTTTTGTCAGCCTATAACAAACATAGAGATAAGCCAATTAGAGAACAAGCAGAAAACATTATTCACTTATTTACCCTTACCAATTTAGGAGCACCTGCTGCTTTCAAGTATTTTGATACAACAATTGATCGTAAAAGATATACCTCAACAAAAGAAGTCTTAGACGCCACCTTAATTCATCAATCAATCACTGGATTGTATGAGACAAGAATTGATTTGTCTCAATTGGGTGGTGATGAAGGGGCTGATCCTAAGAAGAAAAGAAAAGTTGATCCAAAGAAAAAGCGTAAGGTGGATCCTAAGAAAAAGAGAAAGGTTGACTACAAAGACCATGACGGTGATTATAAAGATCATGACATCGACTACAAGGATGACGATGACAAGTGATAATGACTGCAGAGATCCATCGACCTGCCGCCAAGCTAATTCCGGGCGAATTTCTGTCGAGTCATGTAATTAGTTATGTCACGCTTACATTCACGCCCTCCCCCCACATCCGCTCTAACCGAAAAGGAAGGAGTTAGACAACCTGAAGTCTAGGTCCCTATTTATTTTTTTATAGTTATGTTAGTATTAAGAACGTTATTTATATTTCAAATTTTTCTTTTTTTTCTGTACAGACGCGTGTACGCATGTAACATTATACTGAAAACCTTGCTTGAGAAGGTTTTGGGACGCTCGAA

Guide RNAs (includes SNR52 promoter, 20bp protospacer, gRNA scaffold, ENO1 terminator):

Cl_sgRNA_Ade1 (pRB733):

GCATCAAGCTGGTCTCATTGACTTGGCGTCCAGTAACGGCATTCGTTTACCAGCCTCCATTATAAACGCTTCTAGGGCTGTTCCAGAAGATGACGAAGAGGAAAACAGCCTGGCCATGGTTCCTGCTCTAGACTGGGATCTTTGGGGCCCTTTGATTTTCTCCTTGGCTTATTCGGTCACTTTGGGGTTCGCTGCACCCAACTCTCAGACCAATATGGTCTTCTCGGGAACGTTTTCTTTCATCTGGGTGTTTTACTTAATTGGTGGCTTGAACATCCAACTTTTAGGGGGTACAATCTCCTTCTTGTCTGCCATCAGTGCATCAGGATATTCCATGTTTCCTGTCGTGGTTGGTGCCGTGGTGAACACTTTAGCAATCAAATGGAGATGGTTGCGTTTGCTCATTATGTGTTTTCTCACAACTTGGAGCATTTATGCCGCAGGAATGAGCCTACGTTGTCTGGGAGTATTGCCTGGAAGAGTATTTTTGGCCATGTATCCGGTTGCTTTGATGTACACCGTTTTGGCATATCTTACCGTCATAACATAATTGTAACCCGAGGTTTCTAGCACTTAATACATGATGTACTAAAAGGTGCGAGTAATTACGAGGCTACCCATAACTTTAATGAATTAAGCTTGCGACATATGGCATTATTTATTGCTGTGCAAGCCATGAATATGTAAATGTAACCAAATATACCTAGTGAGTTGTGCAATCATGTCTTCTTCAGTACACGTAAACTACCAACATACTTTCAGTGAAGAAAACACAATAACCCAAACAGGCACAATCACAGACGCAGCTAAAACCACCTCATATCTTCGCAGACATTTCTTCATGAAATCACTTACATTTTTCACTACAGGTTGCTCATACTCGCTGAGAAAAAAAAATGTTGTTATTTCATTTGCTGTGGCCCAGTCGGGACGCGCAATGCGCGATCCGGCATGCGGAGGTTCGATTCCTTCGGCGAACAACAAGACTATTGGTATCCTAGGGTTTTAGAGCTAGAAATAGCAAGTTAAAATAAGGCTAGTCCGTTATCAACTTGAAAAAGTGGCACCGAGTCGGTGCTTTTTTTCTCGAGTTTTTTTATCGAGTGTTTAAGGATAATGATAACTGAAGAGAAGAATTAGTTTTGCCGCCACCGCGGGTTTGCCTCTGATTAAATAAAAAAAAGCTGGTGCTTTTTTTTTCTTTTATAGGAACATCTTGAATATATGAACTAATTAAATGATAATTTTTTACCCATCTTTACTCTTAATCACTGAGCTGCAGTCAAAGAAAAAGGGATACAGCACCTGGTGAAGAGATGAACGGAGACTAACTTAGACGCGTTGATTCTTTTTAATTGCACATTTTATTAATCGATGCTAACGTCTATTTACATATATTCTTTAGAGATATTATCTAGGGCTTCAAATAATCTCTGGACAGCAATAAAAGTCTCTTCAAAAGTATTGTATAACGGCAATGGGGCTAATCTGATTACATCTGGTCTTCTTTCGTCACAGATTATAGCATGATCATGCAAGTACGCATTAACTCGTTCCATGACGTTCTTGTCCTTTTCATCGAAATGCGGTTGAAACATAATGGACAATTGACATCCTCTTTCAGCTGGATTCAAAGGAGTTAAAATTTTAAACCCAAATTTGGAGTTTGATGTACTGGATTGTGGTATGTAATACTTGGAATTCGTCAATAGATCCTGTAAAAATTGAGTCAAAGCAACACTTTTTTCACGAAGTTTAGATACTCCACCCACTTTAGCATACACTTCCAATGACGACTTCACAGCAACAACATCAAGAACAGAAGGATTTGACTGTCTGTAAGAAAGAGCCGAGTTTATTGGATCAAACTCTTCTAACATTTTGAATCGTTCTTGGGAGTTATTGCCCCACCAACCAGCTAGTCTAGGAACGAAACTGCTTTTCTTGTTCTCTATGGTGTATTTTTCATGCACAAAAATCCCACCTATGGCTCCAGGTCCCGAGTTTAAATATTTGT

Cl_sgRNA_Ade2 (pRB734):

GCATCAAGCTGGTCTCATTGACTTGGCGTCCAGTAACGGCATTCGTTTACCAGCCTCCATTATAAACGCTTCTAGGGCTGTTCCAGAAGATGACGAAGAGGAAAACAGCCTGGCCATGGTTCCTGCTCTAGACTGGGATCTTTGGGGCCCTTTGATTTTCTCCTTGGCTTATTCGGTCACTTTGGGGTTCGCTGCACCCAACTCTCAGACCAATATGGTCTTCTCGGGAACGTTTTCTTTCATCTGGGTGTTTTACTTAATTGGTGGCTTGAACATCCAACTTTTAGGGGGTACAATCTCCTTCTTGTCTGCCATCAGTGCATCAGGATATTCCATGTTTCCTGTCGTGGTTGGTGCCGTGGTGAACACTTTAGCAATCAAATGGAGATGGTTGCGTTTGCTCATTATGTGTTTTCTCACAACTTGGAGCATTTATGCCGCAGGAATGAGCCTACGTTGTCTGGGAGTATTGCCTGGAAGAGTATTTTTGGCCATGTATCCGGTTGCTTTGATGTACACCGTTTTGGCATATCTTACCGTCATAACATAATTGTAACCCGAGGTTTCTAGCACTTAATACATGATGTACTAAAAGGTGCGAGTAATTACGAGGCTACCCATAACTTTAATGAATTAAGCTTGCGACATATGGCATTATTTATTGCTGTGCAAGCCATGAATATGTAAATGTAACCAAATATACCTAGTGAGTTGTGCAATCATGTCTTCTTCAGTACACGTAAACTACCAACATACTTTCAGTGAAGAAAACACAATAACCCAAACAGGCACAATCACAGACGCAGCTAAAACCACCTCATATCTTCGCAGACATTTCTTCATGAAATCACTTACATTTTTCACTACAGGTTGCTCATACTCGCTGAGAAAAAAAAATGTTGTTATTTCATTTGCTGTGGCCCAGTCGGGACGCGCAATGCGCGATCCGGCATGCGGAGGTTCGATTCCTTCGGCGAACAACGTCAAGTCCTTCCCTGGGTGGTTTTAGAGCTAGAAATAGCAAGTTAAAATAAGGCTAGTCCGTTATCAACTTGAAAAAGTGGCACCGAGTCGGTGCTTTTTTTCTCGAGTTTTTTTATCGAGTGTTTAAGGATAATGATAACTGAAGAGAAGAATTAGTTTTGCCGCCACCGCGGGTTTGCCTCTGATTAAATAAAAAAAAGCTGGTGCTTTTTTTTTCTTTTATAGGAACATCTTGAATATATGAACTAATTAAATGATAATTTTTTACCCATCTTTACTCTTAATCACTGAGCTGCAGTCAAAGAAAAAGGGATACAGCACCTGGTGAAGAGATGAACGGAGACTAACTTAGACGCGTTGATTCTTTTTAATTGCACATTTTATTAATCGATGCTAACGTCTATTTACATATATTCTTTAGAGATATTATCTAGGGCTTCAAATAATCTCTGGACAGCAATAAAAGTCTCTTCAAAAGTATTGTATAACGGCAATGGGGCTAATCTGATTACATCTGGTCTTCTTTCGTCACAGATTATAGCATGATCATGCAAGTACGCATTAACTCGTTCCATGACGTTCTTGTCCTTTTCATCGAAATGCGGTTGAAACATAATGGACAATTGACATCCTCTTTCAGCTGGATTCAAAGGAGTTAAAATTTTAAACCCAAATTTGGAGTTTGATGTACTGGATTGTGGTATGTAATACTTGGAATTCGTCAATAGATCCTGTAAAAATTGAGTCAAAGCAACACTTTTTTCACGAAGTTTAGATACTCCACCCACTTTAGCATACACTTCCAATGACGACTTCACAGCAACAACATCAAGAACAGAAGGATTTGACTGTCTGTAAGAAAGAGCCGAGTTTATTGGATCAAACTCTTCTAACATTTTGAATCGTTCTTGGGAGTTATTGCCCCACCAACCAGCTAGTCTAGGAACGAAACTGCTTTTCTTGTTCTCTATGGTGTATTTTTCATGCACAAAAATCCCACCTATGGCTCCAGGTCCCGAGTTTAAATATTTGT

sgRNA_Ade1 (pRB736):

AAGAAAGAAAGAAAACCAGGAGTGAAAATTAGAAAAGGAAAGGAAAGGAAAAAAAGAAAAATCTGAAAATATATAAAAAAAAATTGTTTCGTTGGCAATAAATCTTGGTGAGAACAGCGACCGAAAGCAAATAAGAACAAAATATGAGTGTATTACGTTGAACAACTAATTAACGTGTGTGTATGGATCTTTTTTTCTTTTTTCTCTTTAACCGACTATAAACAACAAACATTTTTGGGCAGTGCACACACTACTTAATATACACAGCATAAATTACACGATTAGAAACAAATTAGCTTATTAAAATAACCTAATCAAACCGAATATTTTATGGTATTATGAGTAAACTATATAATATAAATAGCACACACCCACAACAACAACAAAGGAAAACTAAAAGGTTTTTTCTTTTTGAAAAGATCGTTTTCTTTATTATTCTCTAGTTTTGACGGCGGCCGCAAGTGATTAGACTTAGTCCGTTCAAATCAAGCACAACTCTGTTCATTGTTTCAACAAGAATTAATTCAAAAACAGGTTCGGTGCATAATTTGCAAAAAAATATTGCAGCTTCTGTGGCTCGAACACAGTACCTCCAGATTTCAGGTTTGAAATACTTCAGTCTGACGCTCTCCCAGATGAGCTAAAGCTGCAATAAGAAAACCCACGCCGGGATTCGAACCCGGAATCCTTTGATTAGAAGTCAAAAGCGATAACCATTTCGCCACGCAGGCCTACTTGATGGGTTTGTAAATGGTCTACTTTTTCAGACCTAACAGAAATTTTAATGAAAGTCATATTCTTATACAATAAAACTGTGTCATAAAAGCAGATATTCGACTTTCGTAGATTATATAGGACCCAAGAACTAAAATTTAATGCCATATTATGCATTTTTAATCTGTAAAAGTGTTGTTTCCAACCTATCACAAGTACGTTCTTGTAACTTGTGTTTGTAGGGTTGCAAATGAATCATAACAACATCTCAACAGAACATGTATAGCAAAGCTTAGTATAAAATCAGTGTTTTGAGAGGCAATCCAAGAATGTTTACATCAAAGTTTCAATAAATATCGACCGAAACTGAAAATCTTTTTAGGTTATTGTTCACTTTTTTGTAAATATTTAAACATTTTTTGGACCTAAAAAAATACAAACACCAATTACGTACCAAGAAGCATCTAATCAACTCCCAGATCACCACTATACATTTAAAAGTCATTGGTCAATAACTATACTCGAGTATTGCCTCATCAAAGAAACAATCAAATATTATAGATACTCACTCCATCACGTGATAATTTCACTGGTATGGAAAAGTGGAAAATTTTATAAAAAAAAATTTGATGCCTTTGGCATAGCTGAAACTTCGGCCCAATAGGATTGGAGAATATGTTTTCGCAGCGTTCTTACAATTAAATTGTGGTGGAAGTTCGAGACTTGCGTAAACTATTTTTAATTTGAAGACTATTGGTATCCTAGGGTTTTAGAGCTAGAAATAGCAAGTTAAAATAAGGCTAGTCCGTTATCAACTTGAAAAAGTGGCACCGAGTCGGTGCTTTTTTTCTCGAGTTTTTTTATCGAGTGTTTAAGGATAATGATAACTGAAGAGAAGAATTAGTTTTGCCGCCACCGCGGGTTTGCCTCTGATTAAATAAAAAAAAGCTGGTGCTTTTTTTTTCTTTTATAGGAACATCTTGAATATATGAACTAATTAAATGATAATTTTTTACCCATCTTTACTCTTAATCACTGAGCTGCAGTCAAAGAAAAAGGGATACAGCACCTGGTGAAGAGATGAACGGAGACTAACTTAGACGCGTTGATTCTTTTTAATTGCACATTTTATTAATCGATGCTAACGTCTATTTACATATATTCTTTAGAGATATTATCTAGGGCTTCAAATAATCTCTGGACAGCAATAAAAGTCTCTTCAAAAGTATTGTATAACGGCAATGGGGCTAATCTGATTACATCTGGTCTTCTTTCGTCACAGATTATAGCATGATCATGCAAGTACGCATTAACTCGTTCCATGACGTTCTTGTCCTTTTCATCGAAATGCGGTTGAAACATAATGGACAATTGACATCCTCTTTCAGCTGGATTCAAAGGAGTTAAAATTTTAAACCCAAATTTGGAGTTTGATGTACTGGATTGTGGTATGTAATACTTGGAATTCGTCAATAGATCCTGTAAAAATTGAGTCAAAGCAACACTTTTTTCACGAAGTTTAGATACTCCACCCACTTTAGCATACACTTCCAATGACGACTTCACAGCAACAACATCAAGAACAGAAGGATTTGACTGTCTGTAAGAAAGAGCCGAGTTTATTGGATCAAACTCTTCTAACATTTTGAATCGTTCTTGGGAGTTATTGCCCCACCAACCAGCTAGTCTAGGAACGAAACTGCTTTTCTTGTTCTCTATGGTGTATTTTTCATGCACAAAAATCCCACCTATGGCTCCAGGTCCCGAGTTTAAATATTTGT

sgRNA_Ade2 (pRB737):

AAGAAAGAAAGAAAACCAGGAGTGAAAATTAGAAAAGGAAAGGAAAGGAAAAAAAGAAAAATCTGAAAATATATAAAAAAAAATTGTTTCGTTGGCAATAAATCTTGGTGAGAACAGCGACCGAAAGCAAATAAGAACAAAATATGAGTGTATTACGTTGAACAACTAATTAACGTGTGTGTATGGATCTTTTTTTCTTTTTTCTCTTTAACCGACTATAAACAACAAACATTTTTGGGCAGTGCACACACTACTTAATATACACAGCATAAATTACACGATTAGAAACAAATTAGCTTATTAAAATAACCTAATCAAACCGAATATTTTATGGTATTATGAGTAAACTATATAATATAAATAGCACACACCCACAACAACAACAAAGGAAAACTAAAAGGTTTTTTCTTTTTGAAAAGATCGTTTTCTTTATTATTCTCTAGTTTTGACGGCGGCCGCAAGTGATTAGACTTAGTCCGTTCAAATCAAGCACAACTCTGTTCATTGTTTCAACAAGAATTAATTCAAAAACAGGTTCGGTGCATAATTTGCAAAAAAATATTGCAGCTTCTGTGGCTCGAACACAGTACCTCCAGATTTCAGGTTTGAAATACTTCAGTCTGACGCTCTCCCAGATGAGCTAAAGCTGCAATAAGAAAACCCACGCCGGGATTCGAACCCGGAATCCTTTGATTAGAAGTCAAAAGCGATAACCATTTCGCCACGCAGGCCTACTTGATGGGTTTGTAAATGGTCTACTTTTTCAGACCTAACAGAAATTTTAATGAAAGTCATATTCTTATACAATAAAACTGTGTCATAAAAGCAGATATTCGACTTTCGTAGATTATATAGGACCCAAGAACTAAAATTTAATGCCATATTATGCATTTTTAATCTGTAAAAGTGTTGTTTCCAACCTATCACAAGTACGTTCTTGTAACTTGTGTTTGTAGGGTTGCAAATGAATCATAACAACATCTCAACAGAACATGTATAGCAAAGCTTAGTATAAAATCAGTGTTTTGAGAGGCAATCCAAGAATGTTTACATCAAAGTTTCAATAAATATCGACCGAAACTGAAAATCTTTTTAGGTTATTGTTCACTTTTTTGTAAATATTTAAACATTTTTTGGACCTAAAAAAATACAAACACCAATTACGTACCAAGAAGCATCTAATCAACTCCCAGATCACCACTATACATTTAAAAGTCATTGGTCAATAACTATACTCGAGTATTGCCTCATCAAAGAAACAATCAAATATTATAGATACTCACTCCATCACGTGATAATTTCACTGGTATGGAAAAGTGGAAAATTTTATAAAAAAAAATTTGATGCCTTTGGCATAGCTGAAACTTCGGCCCAATAGGATTGGAGAATATGTTTTCGCAGCGTTCTTACAATTAAATTGTGGTGGAAGTTCGAGACTTGCGTAAACTATTTTTAATTTGGTCAAGTCCTTCCCTGGGTGGTTTTAGAGCTAGAAATAGCAAGTTAAAATAAGGCTAGTCCGTTATCAACTTGAAAAAGTGGCACCGAGTCGGTGCTTTTTTTCTCGAGTTTTTTTATCGAGTGTTTAAGGATAATGATAACTGAAGAGAAGAATTAGTTTTGCCGCCACCGCGGGTTTGCCTCTGATTAAATAAAAAAAAGCTGGTGCTTTTTTTTTCTTTTATAGGAACATCTTGAATATATGAACTAATTAAATGATAATTTTTTACCCATCTTTACTCTTAATCACTGAGCTGCAGTCAAAGAAAAAGGGATACAGCACCTGGTGAAGAGATGAACGGAGACTAACTTAGACGCGTTGATTCTTTTTAATTGCACATTTTATTAATCGATGCTAACGTCTATTTACATATATTCTTTAGAGATATTATCTAGGGCTTCAAATAATCTCTGGACAGCAATAAAAGTCTCTTCAAAAGTATTGTATAACGGCAATGGGGCTAATCTGATTACATCTGGTCTTCTTTCGTCACAGATTATAGCATGATCATGCAAGTACGCATTAACTCGTTCCATGACGTTCTTGTCCTTTTCATCGAAATGCGGTTGAAACATAATGGACAATTGACATCCTCTTTCAGCTGGATTCAAAGGAGTTAAAATTTTAAACCCAAATTTGGAGTTTGATGTACTGGATTGTGGTATGTAATACTTGGAATTCGTCAATAGATCCTGTAAAAATTGAGTCAAAGCAACACTTTTTTCACGAAGTTTAGATACTCCACCCACTTTAGCATACACTTCCAATGACGACTTCACAGCAACAACATCAAGAACAGAAGGATTTGACTGTCTGTAAGAAAGAGCCGAGTTTATTGGATCAAACTCTTCTAACATTTTGAATCGTTCTTGGGAGTTATTGCCCCACCAACCAGCTAGTCTAGGAACGAAACTGCTTTTCTTGTTCTCTATGGTGTATTTTTCATGCACAAAAATCCCACCTATGGCTCCAGGTCCCGAGTTTAAATATTTGT

Deletion constructs

Long flank ADE2 deletion construct (3780 bp: 1 kb ADE2 flanks + NAT marker; pRB620):

GTCACATAAAGGGGACAGAGAAAGAAAGGTATTATACAATATCCTTCAATTAAGTTTCAGTAAACTGGGGGAAGAGATTCACTCGCTAAAATCGTATTTAATTGTCTTTTCCATGCACACACTATGCATTGGAGCTAATTATGTCTTCTGATATTAGTACTAGAAGTAGATACATGGTAGAAGGATAAGGAATTCAGCCTTTTTATGTTCAGCTATGTGTACCTATGGATTTGTAGAAAGTGTCTTAGAAGTTGGAGAGTTGCTTGTCCTAAGAACATGTCAGAGATAGATGAGATGTATACAGGTTACTGGGATGAGATGAGAAAAATTACCTACACCACATCTGATTGCATCGAAAATGGCAAAATCATTGAGGTCCAAGCCTAAGTTGAGAGCAAAGTCTATCAAGAGGAAAAATGAGTTTTCCAAGTTTGTTGACTCCAGAAATGAAAGATTGGCTCAAAGAATGAAGGTCAATTTAGAAAAGCAAAAGGAAGAAACTATGGAAGAAGACCCCGTCGAAACCAACGAGCCCAAAGAAGAAAAGAAAATCAGCACTTCCGGATGGAGACAAACAAGCAGACAAAAATTGAAACAGAAGAAGAAAAACAAGAAGAATGTTACCAAGTTTTAACTTGGCTTATGCCATCACTTACTTCTGTATACTTAGATGAATAAAAACATGCACGGACATACGATTTGGGGACGGAAGCATATTTACTTGGGGGCCAAATTAAGGCCATTTTATAGTTAGATTACTTGTTTTAAAAATTGACGCCAACGTATCATTGTCGAGAACGTACTATCAGCTCTCAGATGGCCCTGCATCAAAATATTGAAGTCTTGACTCCGATCGCGATAATATAATTGCACAATTGCGCTCCTCCATATTCGTGGAACATCATAAAATGTCGAGCGTCAAAACTAGAGAATAATAAAGAAAACGATCTTTTCAAAAAGAAAAAACCTTTTAGTTTTCCTTTGTTGTTGTTGTGGGTGTGTGCTATTTATATTATATAGTTTACTCATAATACCATAAAATATTCGGTTTGATTAGGTTATTTTAATAAGCTAATTTGTTTCTAATCGTGTAATTTATGCTGTGTATATTAAGTAGTGTGTGCACTGCCCAAAAATGTTTGTTGTTTATAGTCGGTTAAAGAGAAAAAAGAAAAAAAGATCCATACACACACGTTAATTAGTTGTTCAACGTAATACACTCATATTTTGTTCTTATTTGCTTTCGGTCGCTGTTCTCACCAAGATTTATTGCCAACGAAACAATTTTTTTTTATATATTTTCAGATTTTTCTTTTTTTCCTTTCCTTTCCTTTTCTAATTTTCACTCCTGGTTTTCTTTCTTTCTTAGAAACATTATCTCGATATTAATATTAAAAAAATATAATCATTCAAAATGGACGGTGGTATGTTTTAGTTTAGCTTCAATTCTAATTGATTGATTAATCAGTTGATTGGTTTCAATATGACAAATGGGTAGGGTGGGAAAACTTCATTTTCAATTCAGATCAAACTTTTTTGTTGTCGACATAATATTTCTCGTTTGGGATGTTACTGTCACATTAATAATACACACACATCAGCTTATAATTTTGAAAGTAATTTATCAGATATGTTGTGACGATCAATGGAAATGGCTAACTTCAATGTATCTGTTCTTCCCCTTTTTCAAAGTTCACGTTTTTTGATTGATTGATTGATCTGTCGGCAGTGGTTTCAAAACCATTCGGTGAGTAATCCTATCAATCAATGTTACGACAAAAGGCTCAATATTCAAAATTGCAATGTTTTATGTTTTCCTACGTGTACTTGTGCAAGGCAATTGATTCAACATTGCTTTTGGTGTTTGACGAGTTTCTAGTTTGGACTTGTGTTGTTATCTGGGCTATACAGATTTCCCGGCTCACTATGAATTTTTTTTTTCGACGCTCAGTGCACACAACTATAAACAACACAAACACAAACACAGCAAGAAAAAAAAAAAACGAACATTGAATTGAAACCAAGCCAACTGAAAAATTCCTTATTTAAATGACTGTCATACTAACCCATTTTTATAGAAGAAGTTGCTGCTTTAGTTATCGATAACGGTTCTCATATGAAAATTTCGGTGATCCCTGAGCAGGTGGCGGAAACATTGGATGCTGAGAACCATTTCATTGTTCGTGAAGTGTTCGATGTGCACCTATCCGACCAAGGCTTTGAACTATCTACCAGAAGTGTGAGCCCCTACCGGAAGGATTACATCTCGGATGATGACTCTGATGAAGACTCTGCTTGCTATGGCGCATTCATCGACCAAGAGCTTGTCGGGAAGATTGAACTCAACTCAACATGGAACGATCTAGCCTCTATCGAACACATTGTTGTGTCGCACACGCACCGAGGCAAAGGAGTCGCGCACAGTCTCATCGAATTTGCGAAAAAGTGGGCACTAAGCAGACAGCTCCTTGGCATACGATTAGAGACACAAACGAACAATGTACCTGCCTGCAATTTGTACGCAAAATGTGGCTTTACTCTCGGCGGCATTGACCTCTTCACGTATAAAACTAGACCTCAAGTCTCGAACGAAACAGCGATGTACTGGTACTGGTTCTCGGGAGCACAGGATGACGCCTAACATATGTGAAGTGTGAAGGGGGAGATTTTCACTTTATTAGATTTGTATATATGTATAATAAATAAATAAATAAGTTAAATAAATAATTAGATAAGGGTGGTAATTATTACTATTTACAATCAAAGGTGGTCCTGCAGGAATGGAAGCAGTTGGTTACGAGAAATACTAGTAATCTAATACTGAATGATTGTTTGACGAGTTATATTTGTACAAGTTTAACAGGGAAAGAGCGTAGGAAATCAACCCTTCGGATACCCGGGGCAATTTAGTTTGAGACATATAGGTAGGGAACGACAAGATGTAGGAACTATTGAAGGACTGGCACAATAATTATTTAGTAATATTGGCCGGCAAAAGGAGCGTCTTCAGGATTACTCCTGACTGATGTTAAATCAACATTAGCTGATGGGTGGTAGATTGGGTTGGCGAATTGAGCATATGGAACATTGACGGGAAGCTGGCGGACTCCACCGTAATTCGGGTTATTCATATGCGCCAAGTCCACCTCTGGTTGATTTGGTGGTACTTGATAAAGTGGATACTCATGAACAACATGAGAAGGACGTTGTACATTGTATGTTTGGTAAGTCTGAGGACCAGCTCCAAACTTGTTGTGGTTATCAGGGCCACCTGTAACGTTACTCAAGGATTGTGGTAGTAATATTCTTGCAGTGGAGGAAACAGCCTGTGTAATCTCATCGACTCTAGAAAATTCCCAGAGAAGAACGGCCTTGATCTTATGCTTGGGAATTGCAGACATTGGTGCTTCGGAGCTGTTATAGATGACTTGTTTCACAGTAACCCCCTTAAATGCTGCATCCAGACAAGTAGGCTGTTGGAGCAACTGGAGGAAAAAACATTTCCAGAACTTGAGTAAAAAAGGCTGGGTTGTATTAACTTGAAAGTCATCTTCATTAACTTTGAGAACTTCATTACCAAATGAATAGACAGTGGTAAAGCTCGAAAGATTTGAATTAGGCGAGGAGTACTCAAGCATATAACTGGTTCTCAATCCATTATCGATGGAGTAATCATCCGGTTTTAAGAACGGGTTAAATATTCTTACCATATTGTGGAATACTGGTACAGGCAAATTGGCGAAGTCTTCTAAGCCAGGAAACCTGTTCGAAATGGCTGCA

Short flank ADE2 deletion construct (4382 bp: 80 bp ADE2 flanks + flippable NAT marker):

GACTCCGATCGCGATAATATAATTGCACAATTGCGCTCCTCCATATTCGTGGAAGATATACTTGCACACATCTAACAAAAGTACCGGGCCCCCCCTCGAGGAAGTTCCTATACTTTCTAGAGAATAGGAACTTCGGATCCAATAATGATTGGTTTGATATTTTTGTCTAGTACCATCTGTACCATTACACTTAAATTATCTTTATATCTGTCTAACTCGACTGTCTGGATTTCATTGATGTAGTCGTATGCATCGTTAGTTCCAAAAAATATTGTCATCAATTTGATATTGGTTTCCGACTCTAAAATTTTTGGAAGAATTTGTCTAGCGTGCTCTGAGTTGTAGCCACTGAAACCACGGTTAATAACATCCAATTTTCGGATATACACATTCTGTAATGCTGGATGAAAGCCATACTGGGTACAACTAAACTGGGTGATGGAGTCACCGAACAACACAAATTTACCGTATTCCATGATTGCTATGGTTGAGAATTTTTTTTTTTTCTTGTCCCACGCCATTTTTCAAATTATGCAGTTGAGAATGTTAGTTTTTGTGTACACCCCGTTCGCTGAATATTTCGGAATAATTCAAAGATTGGGGAGTGGGGGAGGCGATAGACGAAGACACGGTATAAAAATGGGCAAAATTTTCCCCAACTTTTTGCAGTGGTTTAACTAATAATCGTCGACATGCCACAATTTGATATATTATGTAAAACACCACCTAAGGTGCTTGTTCGTCAGTTTGTGGAAAGGTTTGAAAGACCTTCAGGTGAGAAAATAGCATTATGTGCTGCTGAACTAACCTATTTATGTTGGATGATTACACATAACGGAACAGCAATCAAGAGAGCCACATTCATGAGCTATAATACTATCATAAGCAATTCGTTGAGTTTCGATATTGTCAATAAATCACTCCAGTTTAAATACAAGACGCAAAAAGCAACAATTTTGGAAGCCTCATTAAAGAAATTGATTCCTGCTTGGGAATTTACAATTATTCCTTACTATGGACAAAAACATCAATCTGATATCACTGATATTGTAAGTAGTTTGCAATTACAGTTCGAATCATCGGAAGAAGCAGATAAGGGAAATAGCCACAGTAAAAAAATGCTTAAAGCACTTCTAAGTGAGGGTGAAAGCATCTGGGAGATCACTGAGAAAATACTAAATTCGTTTGAGTATACTTCGAGATTTACAAAAACAAAAACTTTATACCAATTCCTCTTCCTAGCTACTTTCATCAATTGTGGAAGATTCAGCGATATTAAGAACGTTGATCCGAAATCATTTAAATTAGTCCAAAATAAGTATTTGGGAGTAATAATCCAGTGTTTAGTGACAGAGACAAAGACAAGCGTTAGTAGGCACATATACTTCTTTAGCGCAAGGGGTAGGATCGATCCACTTGTATATTTGGATGAATTTTTGAGGAATTCTGAACCAGTCCTAAAACGAGTAAATAGGACCGGCAATTCTTCAAGCAATAAACAGGAATACCAATTATTAAAAGATAACTTAGTCAGATCGTACAATAAAGCTTTGAAGAAAAATGCGCCTTATTCAATCTTTGCTATAAAAAATGGCCCAAAATCTCACATTGGAAGACATTTGATGACCTCATTTCTTTCAATGAAGGGCCTAACGGAGTTGACTAATGTTGTGGGAAATTGGAGCGATAAGCGTGCTTCTGCCGTGGCCAGGACAACGTATACTCATCAGATAACAGCAATACCTGATCACTACTTCGCACTAGTTTCTCGGTACTATGCATATGATCCAATATCAAAGGAAATGATAGCATTGAAGGATGAGACTAATCCAATTGAGGAGTGGCAGCATATAGAACAGCTAAAGGGTAGTGCTGAAGGAAGCATACGATACCCCGCATGGAATGGGATAATATCACAGGAGGTACTAGACTACCTTTCATCCTACATAAATAGACGCATATAAGAGTGAAATTCTGGAAATCTGGAAATCTGGTTTTGTATTCTTGTTATTCTTCTTTTTGTTATTACATATATAACTTGTTACTTTTTTAAAAAAATCTTTGTATATTTTATAAATATATAAAACTAAATTTAAGAAAAAGAGAAAAATGTTTTATTTGAGAGATTGAAATTTTACTTGAATTTAGCTTAGCTTTTATAAAGTATTATTATGTAAAAAAACAAAACAAATATACATTAAAAAGTTAAGACTATAAAATAGCCACCCAAGGCATTTCTATATCTTGTTGTTGTTGTTTTCATCTTCTGTATCAGAGGAACTTATTTTATTATTTTCGTCACGGGTATTTTCTCTTGTTTGATGATTCATCCCATTCATTCCATCATAAAATGTCGAGCGTCAAAACTAGAGAATAATAAAGAAAACGATCTTTTCAAAAAGAAAAAACCTTTTAGTTTTCCTTTGTTGTTGTTGTGGGTGTGTGCTATTTATATTATATAGTTTACTCATAATACCATAAAATATTCGGTTTGATTAGGTTATTTTAATAAGCTAATTTGTTTCTAATCGTGTAATTTATGCTGTGTATATTAAGTAGTGTGTGCACTGCCCAAAAATGTTTGTTGTTTATAGTCGGTTAAAGAGAAAAAAGAAAAAAAGATCCATACACACACGTTAATTAGTTGTTCAACGTAATACACTCATATTTTGTTCTTATTTGCTTTCGGTCGCTGTTCTCACCAAGATTTATTGCCAACGAAACAATTTTTTTTTATATATTTTCAGATTTTTCTTTTTTTCCTTTCCTTTCCTTTTCTAATTTTCACTCCTGGTTTTCTTTCTTTCTTAGAAACATTATCTCGATATTAATATTAAAAAAATATAATCATTCAAAATGGACGGTGGTATGTTTTAGTTTAGCTTCAATTCTAATTGATTGATTAATCAGTTGATTGGTTTCAATATGACAAATGGGTAGGGTGGGAAAACTTCATTTTCAATTCAGATCAAACTTTTTTGTTGTCGACATAATATTTCTCGTTTGGGATGTTACTGTCACATTAATAATACACACACATCAGCTTATAATTTTGAAAGTAATTTATCAGATATGTTGTGACGATCAATGGAAATGGCTAACTTCAATGTATCTGTTCTTCCCCTTTTTCAAAGTTCACGTTTTTTGATTGATTGATTGATCTGTCGGCAGTGGTTTCAAAACCATTCGGTGAGTAATCCTATCAATCAATGTTACGACAAAAGGCTCAATATTCAAAATTGCAATGTTTTATGTTTTCCTACGTGTACTTGTGCAAGGCAATTGATTCAACATTGCTTTTGGTGTTTGACGAGTTTCTAGTTTGGACTTGTGTTGTTATCTGGGCTATACAGATTTCCCGGCTCACTATGAATTTTTTTTTTCGACGCTCAGTGCACACAACTATAAACAACACAAACACAAACACAGCAAGAAAAAAAAAAAACGAACATTGAATTGAAACCAAGCCAACTGAAAAATTCCTTATTTAAATGACTGTCATACTAACCCATTTTTATAGAAGAAGTTGCTGCTTTAGTTATCGATAACGGTTCTCATATGAAAATTTCGGTGATCCCTGAGCAGGTGGCGGAAACATTGGATGCTGAGAACCATTTCATTGTTCGTGAAGTGTTCGATGTGCACCTATCCGACCAAGGCTTTGAACTATCTACCAGAAGTGTGAGCCCCTACCGGAAGGATTACATCTCGGATGATGACTCTGATGAAGACTCTGCTTGCTATGGCGCATTCATCGACCAAGAGCTTGTCGGGAAGATTGAACTCAACTCAACATGGAACGATCTAGCCTCTATCGAACACATTGTTGTGTCGCACACGCACCGAGGCAAAGGAGTCGCGCACAGTCTCATCGAATTTGCGAAAAAGTGGGCACTAAGCAGACAGCTCCTTGGCATACGATTAGAGACACAAACGAACAATGTACCTGCCTGCAATTTGTACGCAAAATGTGGCTTTACTCTCGGCGGCATTGACCTCTTCACGTATAAAACTAGACCTCAAGTCTCGAACGAAACAGCGATGTACTGGTACTGGTTCTCGGGAGCACAGGATGACGCCTAACATATGTGAAGTGTGAAGGGGGAGATTTTCACTTTATTAGATTTGTATATATGTATAATAAATAAATAAATAAGTTAAATAAATAATTAGATAAGGGTGGTAATTATTACTATTTACAATCAAAGGTGGTCCTGCAGGAAGTTCCTATACTTTCTAGAGAATAGGAACTTCAGATCCACTAGTTCTAGAGCGGCCGCCACCGCGGTGGAGCTCCAGCTTTAATCTAATACTGAATGATTGTTTGACGAGTTATATTTGTACAAGTTTAACAGGGAAAGAGCGTAGGAAATCAACCCTTC

Non-homologous end joining sequences (sequenced with primer 20: Cl ADE2 ORF NHEJ check/R):

Colony 1 (protospacer, PAM)

AAGAAAATCAGCACTTCCGGATGGAGACAAACAAGCAGACAAAAATTGAAACAGAAGAAGAAAAACAAGAAGAATGTTACCAAGTTTTAACTTGGCTTATGCCATCACTTACTTCTGTATACTTAGATGAATAAAAACATGCACGGACATACGATTTGGGGACGGAAGCATATTTACTTGGGGGCCAAATTAAGGCCATTTTATAGTTAGATTACTTGTTTTAAAAATTGACGCCAACGTATCATTGTCGAGAACGTACTATCAGCTCTCAGATGGCCCTGCATCAAAATATTGAAGTCTTGACTCCGATCGCGATAATATAATTGCACAATTGCGCTCCTCCATATTCGTGGAAGATATACTTGCACACATCTAACAAAAATGGACGGTAAGACTATTGGTATCCAGGCGGTGGTCAGTTGGGCCGTATGCTTGTTGAAGCAGCTCACAGGCTCAACGTTAAAACCATCATTTTAGACCAGCCCAATTCTCCTGCCAAGCAGATTAATGCTTTGGATGAGCATGTTGATGGTTCTTTTACCGATTTGGATTCTATCACCAAGCTTGCTAAAAAGTGTGATGTGTTAACCGTTGAAATCGAACATGTGGATGTGGAGGCGTTGAAGTCTGTCTCGAAGAGCTTGAACATTCCGATTTACCCTCTTCCCGAAACCATCCGTCTCATCCAAGATAAGTACTTGCAAAAAACACAT

Colony 8 (protospacer, PAM)

AAGAAAATCAGCACTTCCGGATGGAGACGAACAAGCAGACAAAAATTGAAACAGAAGAAGAAAAACAAGAAGAATGTTACCAAGTTTTAACTTGGCTTATGCCATCACTTACTTCTGTATACTTAGATGAATAAAAACATGCACGGACATACGATTTGGGGACGGAAGCATATTTACTTGGGGGCCAAATTAAGGCCATTTTATAGTTAGATTACTTGTTTTAAAAATTGACGCCAACGTATCATTGTCGAGAACGTACTATCAGCTCTCAGATGGCCCTGCATCAAAATATTGAAGTCTTGACTCCGATCGCGATAATATAATTGCACAATTGCGCTCCTCCATATTCGTGGAAGATATACTTGCACACATCTAACAAAAATGGACGGTAAGACTATTGGTATCAGGCGGTGGTCAGTTGGGCCGTATGCTTGTTGAAGCAGCTCACAGGCTCAACGTTAAAACCATCATTTTAGACCAGCCCAATTCTCCTGCCAAGCAGATTAATGCTTTGGATGAGCATGTTGATGGTTCTTTTACCGATTTGGATTCTATCACCAAGCTTGCTAAAAAGTGTGATGTGTTAACCGTTGAAATCGAACATGTGGATGTGGAGGCGTTGAAGTCTGTCTCGAAGAGCTTGAACATTCCGATTTACCCTCTTCCCGAAACCATCCGTCTCATCCAAGATAAGTACTTGCAAAAAACACAT

Additional 20 bp protospacer sequences used to target other genes (using Cl_sgRNAs):

| **Target gene_guide#** | **20 bp protospacer** |
| --- | --- |
| Alpha1_guide1 | TCCCAGAGACGCTCTATAAA |
| Alpha1_guide2 | GCCATCAATGCACTTGAAGC |
| Alpha1_guide3 | CTGGCGCTCCTCTGAAATTA |
| UME6_guide1 | TGGAACAAGCACTTCCGATG |
| UME6_guide2 | ACAGAGAGAACGGAGGTGTT |
| UME6_guide3 | TCTTCCAAGTGCATTGCCTT |
| REC8_guide1 | AGTTGCCAAGATCCATGCCA |
